# Heterogeneity in perceptual category learning by high functioning children with autism spectrum disorder

**DOI:** 10.3389/fnint.2015.00042

**Published:** 2015-06-23

**Authors:** Eduardo Mercado, Barbara A. Church, Mariana V. C. Coutinho, Alexander Dovgopoly, Christopher J. Lopata, Jennifer A. Toomey, Marcus L. Thomeer

**Affiliations:** ^1^Department of Psychology, The State University of New YorkBuffalo, NY, USA; ^2^Institute for Autism Research, Canisius College, Buffalo, NYUSA; ^3^Summit Educational ResourcesGetzville, NY, USA

**Keywords:** prototype, hyperspecificity, categorization, autism, Asperger’s, cortical plasticity

## Abstract

Previous research suggests that high functioning (HF) children with autism spectrum disorder (ASD) sometimes have problems learning categories, but often appear to perform normally in categorization tasks. The deficits that individuals with ASD show when learning categories have been attributed to executive dysfunction, general deficits in implicit learning, atypical cognitive strategies, or abnormal perceptual biases and abilities. Several of these psychological explanations for category learning deficits have been associated with neural abnormalities such as cortical underconnectivity. The present study evaluated how well existing neurally based theories account for atypical perceptual category learning shown by HF children with ASD across multiple category learning tasks involving novel, abstract shapes. Consistent with earlier results, children’s performances revealed two distinct patterns of learning and generalization associated with ASD: one was indistinguishable from performance in typically developing children; the other revealed dramatic impairments. These two patterns were evident regardless of training regimen or stimulus set. Surprisingly, some children with ASD showed both patterns. Simulations of perceptual category learning could account for the two observed patterns in terms of differences in neural plasticity. However, no current psychological or neural theory adequately explains why a child with ASD might show such large fluctuations in category learning ability across training conditions or stimulus sets.

## Introduction

Autism spectrum disorder (ASD) is characterized by deficits in communicative and social skills as well as repetitive actions/fixed interests (American Psychiatric Association, 2013). Because these deficits are behaviorally salient and a major source of difficulties faced by individuals with ASD, researchers have often focused on these symptoms when attempting to identify or treat the underlying causes of this disorder (Baron-Cohen, 2002; Volkmar et al., 2004; Dawson et al., 2010; Bishop-Fitzpatrick et al., 2013). Recent neural and behavioral evidence suggests, however, that less obvious dysfunctions in basic learning and perceptual-motor mechanisms may play a greater role in ASD than was previously assumed (Casanova et al., 2002; Rubenstein and Merzenich, 2003; Markram and Markram, 2010; LeBlanc and Fagiolini, 2011; Yizhar et al., 2011; Donnellan et al., 2012; Robledo et al., 2012; Torres et al., 2013). Such difficulties may degrade a child’s ability to learn basic categories and to generalize what they learn (Cohen, 1998; McClelland, 2000; Grossberg and Seidman, 2006; Dovgopoly and Mercado, 2013). Learning to correctly categorize facial, vocal, and body language expressions are important precursors to understanding and using the social cues that guide interactions and communication (Rochat, 1999; Mundy and Neal, 2000; Kuhl, 2004; Quinn et al., 2011; Vouloumanos and Curtin, 2014). Consequently, category learning and generalization deficits may underlie or exacerbate many of the social and communicative deficits seen in ASD.

Research examining category learning and generalization in individuals with ASD has produced mixed findings (Klinger and Dawson, 2001; Molesworth et al., 2005; Bott et al., 2006; Gastgeb et al., 2006, 2012; Molesworth et al., 2008; Church et al., 2010; Vladusich et al., 2010; Soulières et al., 2011; Froehlich et al., 2012; Schipul, 2012). With binary feature categories, Klinger and Dawson (2001) found deficits in the use of prototypes in children with ASD. Since that initial finding, other researchers using the same type of categories have found normal prototype effects in recognition memory and categorization performance (Molesworth et al., 2005; Soulières et al., 2011). However, some individuals with ASD showed clear difficulties in the initial phases of category formation (Bott et al., 2006; Molesworth et al., 2008; Soulières et al., 2011). In research using more complicated visual images, such as faces and random dot patterns (RDPs), the findings are also mixed (Gastgeb et al., 2009, 2012; Church et al., 2010; Vladusich et al., 2010; Soulières et al., 2011; Froehlich et al., 2012; Schipul, 2012). Studies of RDP category learning by children and adults with ASD, and of face categorization by adults, revealed significant abnormalities in both learning and generalization (Gastgeb et al., 2009, 2012; Church et al., 2010, 2015). Other studies of adults with ASD using similar stimuli did not find significant abnormalities in generalization after training with visually complex categories, though most did find significant differences in other measures such as learning rate (LR) and brain adaptation (Vladusich et al., 2010; Soulières et al., 2011; Froehlich et al., 2012; Schipul, 2012; Fiebelkorn et al., 2013).

Past explanations for why individuals with ASD show category learning deficits have focused on differences in perception (Plaisted et al., 1998; O’Riordan and Plaisted, 2001; Mottron et al., 2006), executive dysfunction (Bott et al., 2006), deficient learning mechanisms (Grossberg and Seidman, 2006; Dawson et al., 2008; Schipul et al., 2012; Dovgopoly and Mercado, 2013), and abnormalities in neural processing (McClelland, 2000; Grossberg and Seidman, 2006; Markram and Markram, 2010; Fiebelkorn et al., 2013). ASD is generally associated with difficulties in transferring learning to novel contexts (Lovaas et al., 1979; Plaisted et al., 1998; Klinger and Dawson, 2001; Mottron and Burack, 2006; Klinger et al., 2007; Dawson et al., 2008; Gastgeb et al., 2009), suggesting that some mechanisms that contribute to atypical perceptual category learning in ASD may also affect various other learning and generalization abilities. ASD is also associated with atypical perceptual processing (Spencer et al., 2000; Happe and Frith, 2006; Mottron et al., 2006; Dawson et al., 2008; Mottron et al., 2009), which could affect how categories are formed, as well as how inputs are represented and compared. Given that perceptual processing is strongly experience-dependent (Buonomano and Merzenich, 1998), perceptual abnormalities associated with ASD could result from atypical learning and plasticity mechanisms that affect early perceptual development (LeBlanc and Fagiolini, 2011).

Differences in methods or sample composition across studies could potentially account for why some researchers have found category learning deficits in individuals with ASD whereas others have not. However, mixed findings have also been reported within single studies (Bott et al., 2006; Molesworth et al., 2008; Vladusich et al., 2010; Dovgopoly and Mercado, 2013). Typically, mixed results within studies have been interpreted as suggestive of either different subgroups of children with distinctive neural or cognitive abnormalities, or of individual differences in basic cognitive abilities. For instance, Molesworth et al. (2008) suggested that category learning deficits might be present in a subset of individuals with ASD who have a lower than normal mental age or more severe language processing deficits. Tests of HF children with ASD whose IQ and language abilities were comparable to those of TD children revealed, however, that about half of the HF children with ASD had problems learning RDP-based visual categories (Church et al., 2010, 2015; Dovgopoly and Mercado, 2013). The neural or behavioral factors that make category learning more difficult for a subset of children with ASD thus remain unclear, as measures of IQ, receptive or expressive language abilities, and overall scores and scores on the subtests of the Autism Diagnostic Inventory Revised have not been found to distinguish the subgroups (Church et al., 2015).

The primary goal of the current study was to evaluate the consistency of visual category learning abilities in HF children with ASD when they were trained using several different stimulus sets and training schedules. In each category-learning task, children were first trained through trial and error to classify abstract shapes as either belonging to the category, or as not belonging to that category, and then were tested without feedback on how they classified novel shapes. Previous work has shown that TD children easily perform such tasks, whereas some children with ASD find these tasks difficult to learn (e.g., Church et al., 2010, 2015). A secondary goal of the study was to evaluate how well a connectionist model of category learning could predict the performance of children with ASD.

## Materials and Methods

### Participants

The study sample consisted of 56 HF children with ASD and thirteen TD children; all were between the ages of 7 and 13 years-old. Thirteen of the children with ASD and the thirteen TD children were recruited to participate in a study comparing slight modifications in the training regimens used for category learning (Situation A). Forty-three different HF children with ASD were recruited to participate in two different category learning studies on the same day (Situation B). The data from their standard training conditions (baseline conditions) are included here. The HF children with ASD had a prior clinical diagnosis of Asperger’s disorder (American Psychiatric Association, 2000), autism, or PDD-NOS (pervasive developmental disorder-not otherwise specified). They were recruited from a psychosocial intervention program and all met strict inclusion criteria. Inclusion criteria were a WISC-IV, (Wechsler, 2003) short-form IQ composite > 70 (and a major index score {VCI or PRI}≥ 80); receptive or expressive language score ≥ 80 on the CASL (Carrow-Woolfolk, 1999), and a score meeting ASD criteria on the ADI-R (Rutter et al., 2003). All testing to determine inclusion (WISC-IV, CASL, and ADI-R) was conducted by doctoral-level psychologists and graduate students with advanced training in the specific measures. These structured screening procedures and inclusion criteria have been used in numerous treatment trials and basic studies for HF children with ASD (e.g., Lopata et al., 2012; Thomeer et al., 2012, 2015), as well as prior perceptual studies of HF children with ASD (e.g., Church et al., 2010, 2015). TD children were recruited by the staff running the psychosocial intervention program from a database of children used in previous studies who matched a subset of the HF children with ASD for age, gender, and IQ. There were no significant differences between the HF children with ASD and their matched controls (see **Table 1** for demographic and test score information). None of the HF children with ASD or the TD children had significant visual impairments or acuity problems. Those that wore corrective lenses for minor visual acuity problems were monitored to ensure they used their corrective lenses during all screening testing and the current experiments. Earlier studies of HF children with ASD found no significant differences in visual perceptual acuity from that of TD children (Volker et al., 2010; McDonald et al., 2014). Ten of the HF children with ASD and 10 matched TD children from Situation A also participated in the Church et al. (2010) experiment, and thus had some past experience with the general task, though not with the particular categories, stimuli, and training specifics experienced in this context. For all of the children, at least one custodial parent or guardian signed a written informed parental permission for their child to participate, and the child signed a written informed assent sheet. The parents/guardians and children had the tasks, time commitments, right to withdraw, and risks/benefits explained by one of the primary researchers before they were asked to grant permission/assent. All procedures were conducted in accordance with a protocol approved by the Social and Behavioral Sciences IRB at the University at Buffalo. Results from two of the HF children with ASD were omitted for having more than four missing values, one for patterned responding (left–right alternation), and four participants were dropped randomly to equate conditions used to counterbalance stimulus sets and the order of their presentation in Situation B.

**Table 1:** Demographic characteristics of matched groups^a^.

	ASD (*n* = 13)	Control (*n* = 13)
Characteristic	Mean (SD)	Mean (SD)
Age (years)	10.77 (1.59)	10.85 (1.52)
Parent education (years)	15.88 (1.80)	15.88 (1.74)
WISC-IV Short Form IQ	109.24 (11.07)	112.74 (9.18)
Verbal IQ (VCI)	108.86 (12.25)	108.45 (9.01)
Performance IQ (PRI)	107.65 (11.26)	114.64 (9.76)
CASL		
Expressive language	105.93 (11.33)	–
Receptive language	111.08 (13.06)	–
ADI-R		
QARSI	21.31 (4.50)	–
QAC	15.46 (4.43)	–
RRSB	6.54 (2.54)	–
	*n* (% of total)	*n* (% of total)
Gender		
Male	11 (85.0)	11 (85.0)
Female	2 (15.0)	2 (15.0)
Ethnicity		
Caucasian	11 (85.0)	11 (85.0)
African American	2 (15.0)	2 (15.0)

*^a^WISC-IV, Wechsler Intelligence Scale for Children-4th Edition; CASL, Comprehensive Assessment of Spoken Language; ADI-R, Autism Diagnostic Interview-Revised (QARSI, Qualitative Abnormalities in Reciprocal Social Interactions; QAC, Qualitative Abnormalities in Communication; RRSB, Restricted, Repetitive, and Stereotyped Patterns of Behavior). CASL and ADI-R data only collected for children in the ASD group as a screening measure for the social treatment study*.

### General Procedure

**FIGURE 1:**
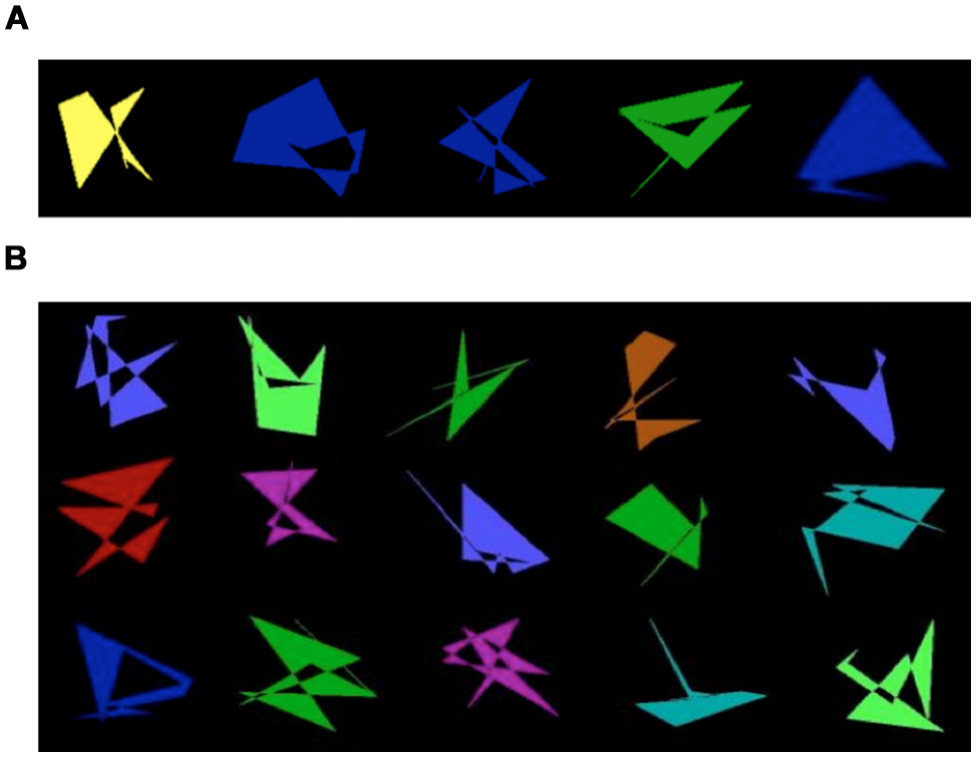
**(A)** Examples of prototype shapes for each category type. **(B)** Examples of non-category shapes created from randomized dot patterns (Random).

Visual stimuli were created using a computer program that generated a single shape (the prototype) and subsequently modified this prototype to generate distorted versions of it (e.g., Smith et al., 2008). The prototype shape was created by selecting nine random dots in a sequential order. The distortions were created by probabilistically moving some or all dots forming the prototype. A low probability of moving the dots resulted in stimuli that strongly resembled the prototype. As the probability of moving the dots increased, the level of distortion increased [e.g., Level 2 (L2), Level 3 (L3), Level 4 (L4), Level 5 (L5), and Level 7 (L7)]. Random stimuli (R) were constructed from sets of dots that were unrelated to the category, and unrelated to each other (see Supplementary Materials for more details about stimuli construction). The dots forming the stimuli were connected with lines; which dots were connected to which was determined by the order of their random selection. The resultant shapes were filled with different colors (see **Figure 1**). Stimuli were of medium brightness and color varied randomly between red, blue, light blue, green, and yellow. An IBM-compatible desktop computer was used for testing in Situation A. IBM compatible laptops were used in Situation B. Stimuli were presented, responses collected and feedback given using DMDX experimental software (Forster and Forster, 2003).

The 26 children from Situation A were tested individually in a quiet room at the lab. They interacted with the experimenter engaging in four computer tasks requiring ∼15 min to complete each task with breaks in between each. They were in the lab for an hour and a half or less during experimental sessions. The 43 children in Situation B were tested individually in groups of four children stationed at different desks with dividers between them at their summer treatment program, and they participated in two studies each having the baseline conditions reported here that lasted ∼12 min. The total experimental session for these children lasted ∼50 min with a short break (∼3 min) between the studies. All tasks had a training phase and a testing phase. The training phase varied depending on the task conditions (described below). The structure of the testing phase was the same across all tasks, varying only in terms of which visual stimuli were presented. Participants learned a new category for each task. **Figure 1A** shows the prototype shape for five categories. All participants received verbal instructions and written reminders about the goal of each task. All children received a cover story that they were going to play a computer game called ghost hunt. In this computer game, the shapes were ghosts and their job on each task was to hunt down the ghosts of a particular type. They were directly told that for each task, the ghosts were abstract shapes, and they were looking for the ghosts of a particular kind (e.g., cave ghost, sea ghost, jungle ghost, desert ghost, and castle ghost). They were also told that ghosts of the same kind resemble each other, but any kind of ghost could be any color; so they should not make any decisions based on the color of the ghost. In Situation A, the children completed four tasks using stimulus sets based on prototypes 1 through 4 (described in detail below). The order of the specific tasks and the stimulus sets used for a particular task varied across children. For TD children, the order of particular tasks and the stimulus set used for each task were the same as for their age, sex, and IQ matched child with ASD.

In Situation B, children completed two baseline category-learning tasks (comparable to those learned by children in Situation A), plus two other category-learning tasks that constituted the experimental conditions (which strongly manipulated the type of learning or stimuli that occurred during the training phase, and thus were not comparable to the tasks used in Situation A). Children’s performance on the baseline tasks from Situation B was included in the current analysis. The order of task and stimulus sets used was fully counterbalanced across children in this situation. Also, in Situation B, researchers made observational recordings on a behavioral tally sheet throughout the study to document and quantify behavioral signs of attention, fidgeting, engagement, and frustration. The children were also asked at the end of each session if they liked each game, and whether they thought each of them was difficult and/or boring.

### Tasks

#### A/not A Category Learning Task

In A/not A tasks, the participant must distinguish category members (**Figure 1A**) from non-members (**Figure 1B**) based on their relative similarity to other members. In the version of this task used in the current study, each trial consisted of one shape presented in the middle of a computer screen against a black background (at a visual angle of ∼4.23° in Situation A and 3.13° in Situation B), and two icons presented on the top left and right of the screen. The icon presented on the left was always a red circle with a line through it. The icon presented on the right was either a picture of a cave, a jungle, a desert, a sea, or a castle. The shape and icons remained on the screen until the participant made a response. All shapes were presented in a random order. The program was designed to move to the next trial if the participant took longer than 7 s to respond and the trial was marked as a missing trial. Participants responded to a category member or not by pushing one of two labeled buttons on a keyboard. The button for each response was aligned on the same side as the icon corresponding to the response.

In all conditions, children first experienced a training phase in which they had to decide if each shape was a member of the ghost category. During training, in Situation A, they always experienced 30 shapes in three of the tasks and 60 shapes in one of the tasks (details provided below). In Situation B, during training they always experienced 30 shapes. In all Situations and tasks (training and test), half of the shapes belonged to the prototype-based category and the other half were non-members. For all Situations and training conditions, participants received a short animation of a dancing monkey for each correct response and the shape moved to the icon of the correct answer after a wrong response. Following the training, written instructions appeared explaining that the true ghost hunt was about to begin, and they would no longer receive any feedback. The test phase followed these instructions. Sixty images were always presented in the test phase (five repetitions of the prototype shape, five different L2, five different L3, five different L4, five different L5, five different L7, and 30 different R stimuli). None of the stimuli presented during testing appeared in the training conditions. No feedback regarding the accuracy of responding was given during testing.

#### Variations of the A/not A Category Learning Task

Forty-three HF children with ASD in Situation B were trained using a category learning task that replicated the A/not A training structure originally used by Church et al. (2010). These children were trained and tested with two versions of the standard baseline task, involving four different sets of shapes constructed using the same algorithms as Church et al. (2010). The task involved training with level 3, 5, and 7 distortions of a prototype ghost (five shapes from each distortion level), as well as 15 randomly (R) created shapes as non-members. Thirty-six of these Situation B children were used in the data analyses (two were dropped for missing values, one for patterned responding and four to equate counterbalancing of order and stimulus set).

Thirteen HF children with ASD (none of whom overlapped with the 36 noted above) and 13 TD children from Situation A were trained and tested on four modified versions of the A/not A task used by Church et al. (2010). Each of the four tasks included a different training regimen and a unique stimulus set. All of the modifications were intended to increase the difficulty of the task and to encourage family resemblance averaging. In the “Repeated” version of the task, which served as the baseline for comparison, 30 shapes were presented in which 15 were equally divided between L3, L5, and L7 and the other 15 were R stimuli. This condition replicated the training regimen described above for the Situation B children, except that each stimulus was shown twice during training. For the “High Distortion” version of the task, we increased the proportion of stimuli with high-level distortions. In this task, the stimuli were four L3, five L5, and six L7 shapes; the other 15 were R stimuli. Each stimulus was shown twice. For the “Blurry” task, we reduced the spatial frequency of the stimuli to three different levels (low, medium, and high). There were five L3, five L5, five L7, and 15 R stimuli. The stimuli were equally divided into three types of reduced spatial frequency and each item was presented twice. For the “Unique” task, we doubled the number of different shapes presented. In this condition, there were ten L3, ten L5, and ten L7, and 30 R stimuli. Each stimulus was shown once. All Situation A children completed all four training tasks.

### Data Analyses

Analyses of behavioral data were focused on answering four main questions. First, we wanted to know whether the A Type I/A Type II classification of generalization patterns identified by Dovgopoly and Mercado (2013) would prove to be generally applicable to HF children with ASD in category learning tasks. They had found that HF children with ASD could be divided into meaningful groups for modeling purposes by applying the criterion that any child with ASD who endorsed random stimuli more than 30% of the time fell into the group modeled with slow learning (A Type II), and any children with ASD who endorsed random stimuli less than 30% of the time qualified as a typical learner (A Type I). However, this criterion was determined *post hoc* based on its correspondence to the grouping of generalization patterns revealed by a SOM that was trained with data from all participants (Dovgopoly and Mercado, 2013), and the criterion has only been validated as a means of identifying atypically performing subgroups of children with ASD in one other study (Church et al., 2015). To address the question of whether the A Type I/A Type II distinction will continue to prove generally applicable, we applied this criterion for identifying children of each type to the new sample of HF children with ASD, and compared the generalization profiles associated with identified subgroups. Specifically, any child who endorsed 30% or more of the random shapes during testing was classified as fitting an A Type II profile. This threshold value, based on past behavioral data from the Church et al. (2010) study, provided an objective criterion for partitioning children into subgroups in the current study (as opposed to using an arbitrary, *post hoc* criterion such as splitting children into two equal-sized groups based on their overall task performance).

Second, we wanted to assess not only the applicability of the A Type I/II classification across samples (and using different stimulus sets), but also the stability of classifications within individuals. To address this question, we applied the classification criterion for each version of the A/not A task performed by each child. We then compared classifications across tasks performed by single individuals. All of the children in the study performed at least two versions of the category learning task, and the 26 children in Situation A (13 ASD and 13 TD) performed four versions of the task. Ten of the HF children with ASD that were trained on the four different tasks also participated in the Church et al. (2010) study, making it possible to assess stability in generalization profiles over a period of 2 years in different testing contexts.

Third, we were interested in the stability of generalization profiles across different variants of the A/not A task. Previous studies have examined how variations in stimulus construction or in feedback conditions affect category learning by HF adults with ASD (Vladusich et al., 2010; Gastgeb et al., 2012), but none have looked at whether variations in training regimens affect learning and generalization by HF children with ASD. Because these new variants of the task had not been previously tested with TD children, comparisons were made between groups of children with and without ASD to assess whether atypical generalization was evident across the four different training regimens. A 3 x (4 x 7) mixed factorial design was used with category endorsement as the dependent measure, group (ASD Type I vs. Type II vs. TD) as a between participants variable with three levels, and condition (Repeated, High Distortion, Blurry, Unique), and stimulus type (prototype, L2, L3, L4, L5, L7, and random) serving as the within participant independent variables with 4 and 7 levels, respectively.

Finally, we wanted to determine if any of the individual demographic variables or scores on ASD scales, language, or IQ tests would predict general performance or the percentage of random endorsements that dictate A Type I/A Type II designations.

### Neural Network Simulations

Dovgopoly and Mercado (2013) showed that a simple connectionist model of visual object recognition was able to simulate the performance of TD children learning to classify abstract shapes and could also reproduce atypical generalization patterns observed in groups of HF children with ASD. This model also successfully predicted generalization differences between HF children with ASD and TD children after training with prototypical images (Church et al., 2015). The applicability and value of this computational model for simulating generalization patterns after different training regimens was assessed in the current study by evaluating predictions of the generalization patterns for the four different A/not A category learning task variants not previously simulated by Dovgopoly and Mercado (2013).

The visual images created by Church et al. (2010) provided the basis for the input set used in all neural network (NN) simulations. This set of inputs consisted of five L3, L5, and L7 distortions, as well as 15 random shapes. In addition to these 30 images, 15 novel distorted prototypes and 15 novel random images were created to simulate stimulus sets used in the Unique condition, and 15 novel stimulus representations from both the prototype distortion and random categories were created to simulate the stimuli used in the Blurry training condition. Test images consisted of the prototype shape and five L2, L3, L4, L5, and L7 distortions (all different from the training images), as well as 30 novel random shapes. The images of abstract shapes were converted into matrices representing features within the images (for details, see Dovgopoly and Mercado, 2013). Images from the original Church et al. (2010) study were used as inputs rather than the actual images used in the current experiments in order to better establish that the predictions of the model were not dependent on the particular input set used.

Simulations were conducted using PDPTool (http://www.stanford.edu/group/pdplab/resources.html#pdptool) running in the Matlab R2010a environment, and using customized data-processing scripts written in Matlab, Perl 5.12.2, and Ruby 1.9.2 programming languages. All simulations involved a multilayer NN with 144 input nodes, 144 hidden layer nodes, and 144 output layer nodes [a detailed description of the parameters of this model is provided in Dovgopoly and Mercado (2013)]. Results for each task correspond to the averages of 20 simulations [replicating the methods of Dovgopoly and Mercado (2013)]. In contrast to our earlier simulations, which used unique, randomly generated connection weights for each simulation, a “within-subjects” design was used in the current simulations. Specifically, randomly generated sets of initial weights were used for simulations of HF children with ASD, and another 20 sets were created for the TD simulations. These same initial weights were used for each of the training conditions, controlling for the possibility that idiosyncratic variations in initial weights or order effects might contribute to differences in generalization patterns across the four conditions.

The first step of the simulations was to establish a performance baseline comparable to the generalization pattern of the TD group participants in the Church et al. (2010) study. After reproducing generalization comparable to that of TD children, a single model parameter was adjusted until the performance of the networks approximated the overall group performance of the HF children with ASD. In one set of simulations, a LR parameter was reduced, thereby decreasing the magnitude of changes in weights during each cycle of training. In a second set of simulations, LR was maintained at the same level as in TD simulations, but a NWD term was introduced, degrading the models’ ability to discover an optimal way of dividing up inputs during training by disrupting feedback-based changes to connection weights. Dovgopoly and Mercado (2013) argued that the reduced LR manipulation simulates diminished synaptic plasticity, whereas adding NWD simulates diminished synaptic stability. One of these two model parameters was initially adjusted to emulate TD and ASD group performance in the “Repeated” training condition. The selected parameter values were then kept fixed across other training conditions (i.e., no attempt was made to identify model parameters that optimally fit the observed behavioral patterns across training conditions). For each of the training regimens, individual networks were trained for three epochs. The LR was 5e-005 for TD simulations, whereas ASD group simulations in which the LR was modified used a LR of 2.1e-005. The ASD group simulations with NWD utilized a weight decay of -0.0007 and the same LR as the TD model (in contrast, the weight decay value for the TD model and the reduced LR model was set to zero). All other model parameters were fixed at default values. The same approach to adjusting LR or NWD was also used in an attempt to simulate the generalization patterns shown by the subgroup of HF children with ASD classified as A Type II. However, no parameter settings were found that led to comparable generalization, and ultimately it was necessary to decrease the number of training epochs to simulate the generalization pattern associated with the A Type II profile. Because the generalization patterns of children with an A Type I profile were indistinguishable from those of TD children, no distinctions are made in the following between simulations of performance by these two groups.

## Results

### Evaluations of A Type I/II Generalization Patterns

**FIGURE 2:**
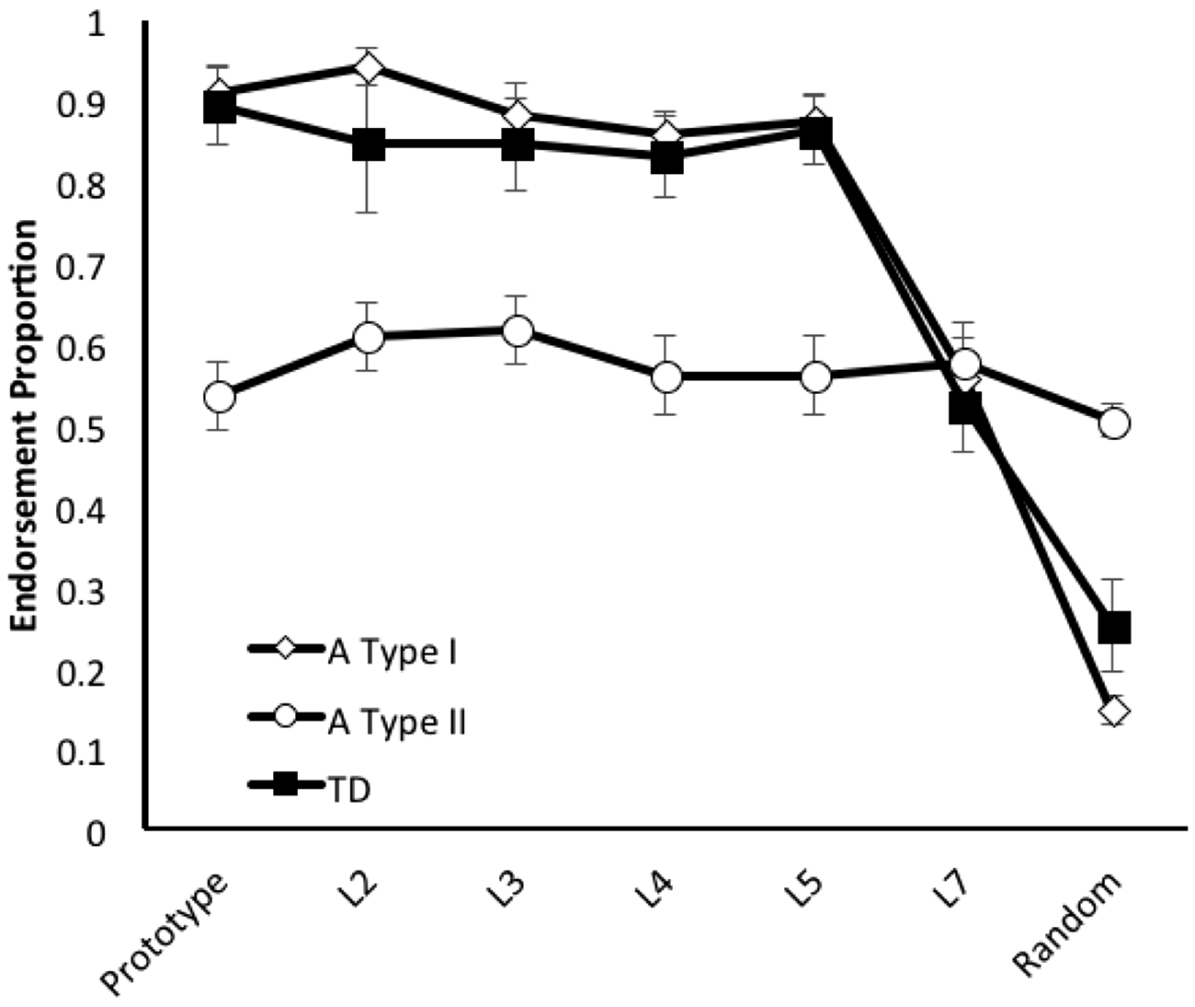
**Average endorsement proportions of Prototype, L2, L3, L4, L5, L7 (increasingly distorted versions of the prototype), and Random images during generalization testing for 62 children (TD = 13; A Type I = 24; A Type II = 25) trained using a category learning task that replicated the A/not A training structure originally used by Church et al. (2010)**. As in the earlier experiment by Church et al. (2010), A Type I generalization patterns were comparable to those shown by TD children, and A Type II patterns revealed much poorer generalization, with more endorsements of low-level distortions than of the prototype shape.

First, in order to assess the generally applicability of the A Type I/II distinction to HF children with ASD in category learning tasks, we applied the pre-established criterion (Dovgopoly and Mercado, 2013; Church et al., 2015) for identifying children of each type to the new sample of children with ASD (any child who endorsed 30% or more of the random shapes during testing was classified as fitting an A Type II profile), and compared the generalization profiles associated with identified subgroups (see **Figure 2**). We then conducted a 3 X 4 X 4 X 7 GLM with Child Type (A Type I, A Type II, and TD) Order of Test (1^st^–4^th^ possible), and Stimulus Set (A–D possible) as the between participant factors and Distortion Type (prototype, L2, L3, L4, L5, L7, and Random) as the within participant factor. There were significant main effects of Child Type, *F*(2,33) = 7.595, *p* = 0.002, $\eta_{p}^{2}$ = 0.315, and Distortion Type, *F*(6,198) = 6.588, *p* < 0.001, $\eta_{p}^{2}$ = 0.116, reflecting the facts that the groups endorsed the category to differing degrees, and stimuli closer to the prototype were generally endorsed more often. There was also a significant interaction between the Child Type and Distortion Type, *F*(12,198) = 4.944, *p* < 0.001, $\eta_{p}^{2}$ = 0.231, reflecting the different pattern of endorsement seen in the A Type II children. No other main effects or interactions approached significance, all *F’*s < 2. The fact that we found no main effects of, or interactions with, order or stimulus set suggests that the results (and group designations) were not affected by a couple of harder stimulus sets, or fatigue, or procedural learning/learning set effects as the children progressed through their tasks^1^. Consistent with the findings of Dovgopoly and Mercado (2013), A Type II children endorsed fewer prototypes, *t*(47) = 7.115, *p* < 0.001, *d* = 1.991, and more non-category members, *t*(47) = 13.682, *p* < 0.001, *d* = -3.839, than A Type I children. The latter should not be surprising because the categories were defined by differences in non-member endorsements. To further determine the independent utility of the A Type distinction, we compared the proportion of endorsements of just the category members (not the criteria for typing) for different A Types and TD children. A Type II children endorsed fewer category members overall than either the A Type I, *t*(47) = 7.724, *p* < 0.001, *d* = 2.160, or TD children, *t*(36) = 4.650, *p* < 0.001, *d* = 1.545. A Type I and TD children endorsed roughly the same proportion of category members, *t* < 1.

As can be seen in **Table 2**, of the children from Situation A who completed four tasks, almost all of the overlapping children showing an A Type II pattern in the Church et al. (2010) study showed an A Type I pattern in the Repeated condition of the current experiment. There were two out of five A Type I children from the previous experiment who switched to A Type II in the Repeated condition, but four out of five of the A Type II children switched to A Type I. This suggests that perhaps more children switch to A Type I as they get older (the small sample precluded any statistical analyses of these trends) indicating that developmental maturation may improve category learning and generalization in HF children with ASD. However, any conclusion must be tentative since age, time, and the testing situation all changed across the 2 year period. In addition to these fluctuations in generalization patterns across experiments, HF children with ASD also showed similar changes across tasks. Sixty-two percent of the HF children with ASD that were tested on four versions of the task (8 out 13) switched their pattern of generalization at least once either across the different tasks (54%, 7 out 13) or across the different experiments (70%, 7 out of 10).

**Table 2:** Number of HF Children with ASD who switch A Types or not across two tasks, four tasks, or 2 years.

A type variability	Four tasks	Two tasks	Two years earlier
Total *N*	13	36	10
Switch A Types	6	13	5
Stay A Type I	3	5	3
Stay A Type II	4	18	2
Multiple switches	1	–	–
More I	2	–	–
More II	4	–	–
Switch from II–I	–	–	4

Of the 36 children from Situation B who completed two versions of the basic task, 64% showed consistent generalization patterns across tasks (5 out of 23 A Type I and 18 out of 23 A Type II), and 36% switched performance profiles between the two versions of the task. There were no patterns of interaction indicating that switching was influenced by order of task or stimulus set used, and analyses of the observational recordings found that behavioral signs of attention, fidgeting, engagement, and self-reports of difficulty, boredom and enjoyment did not predict A Type designation. There were not enough recorded signs of frustration to provide a meaningful analysis.

### Comparisons of Different Types of Training

**FIGURE 3:**
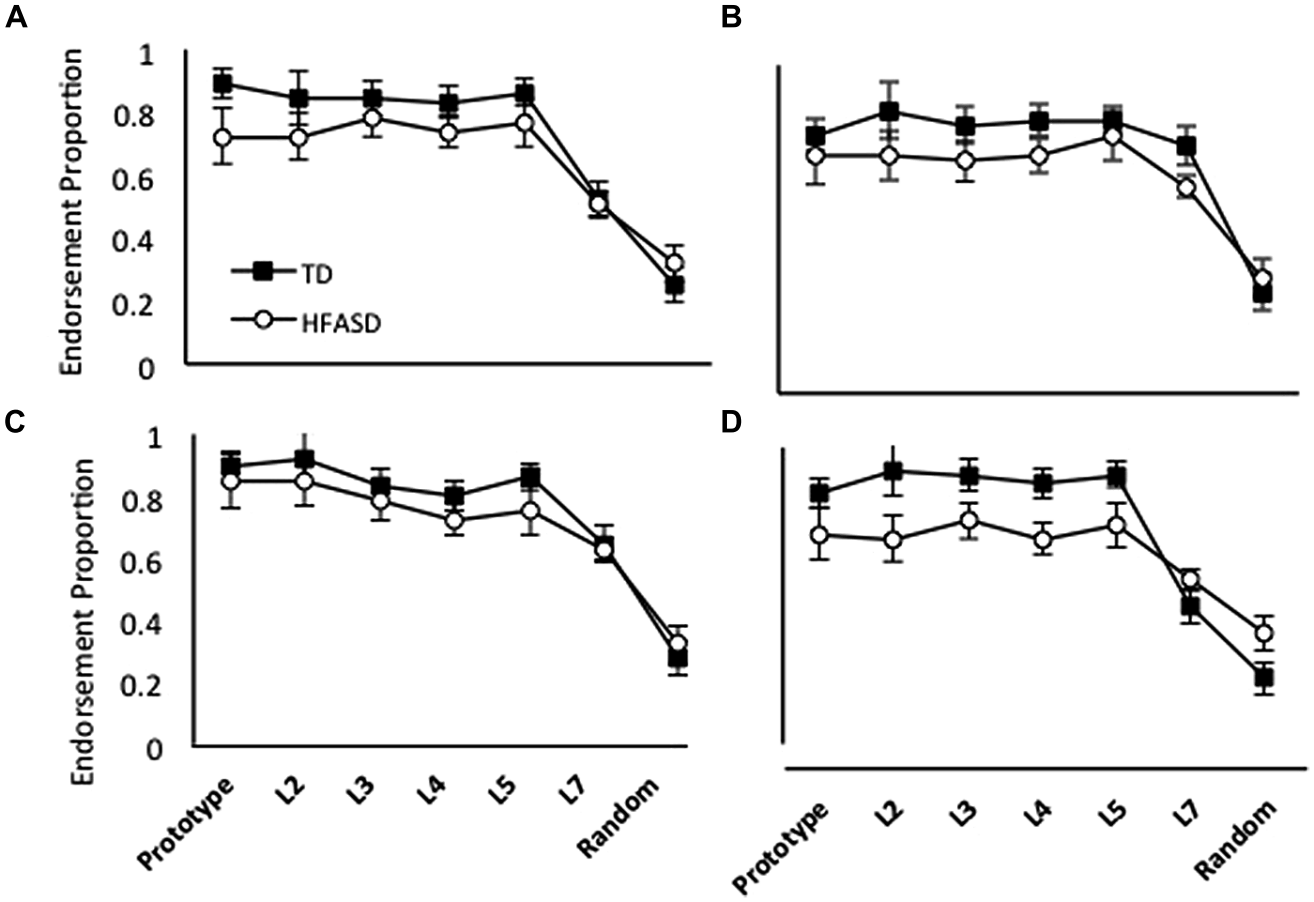
**Average endorsement proportions of Prototype, L2, L3, L4, L5, L7 and Random images during testing for 13 TD children and 13 HF children with ASD in four conditions: **(A)** Repeated; **(B)** High Distortion; **(C)** Blurry; and **(D)** Unique**.

**FIGURE 4:**
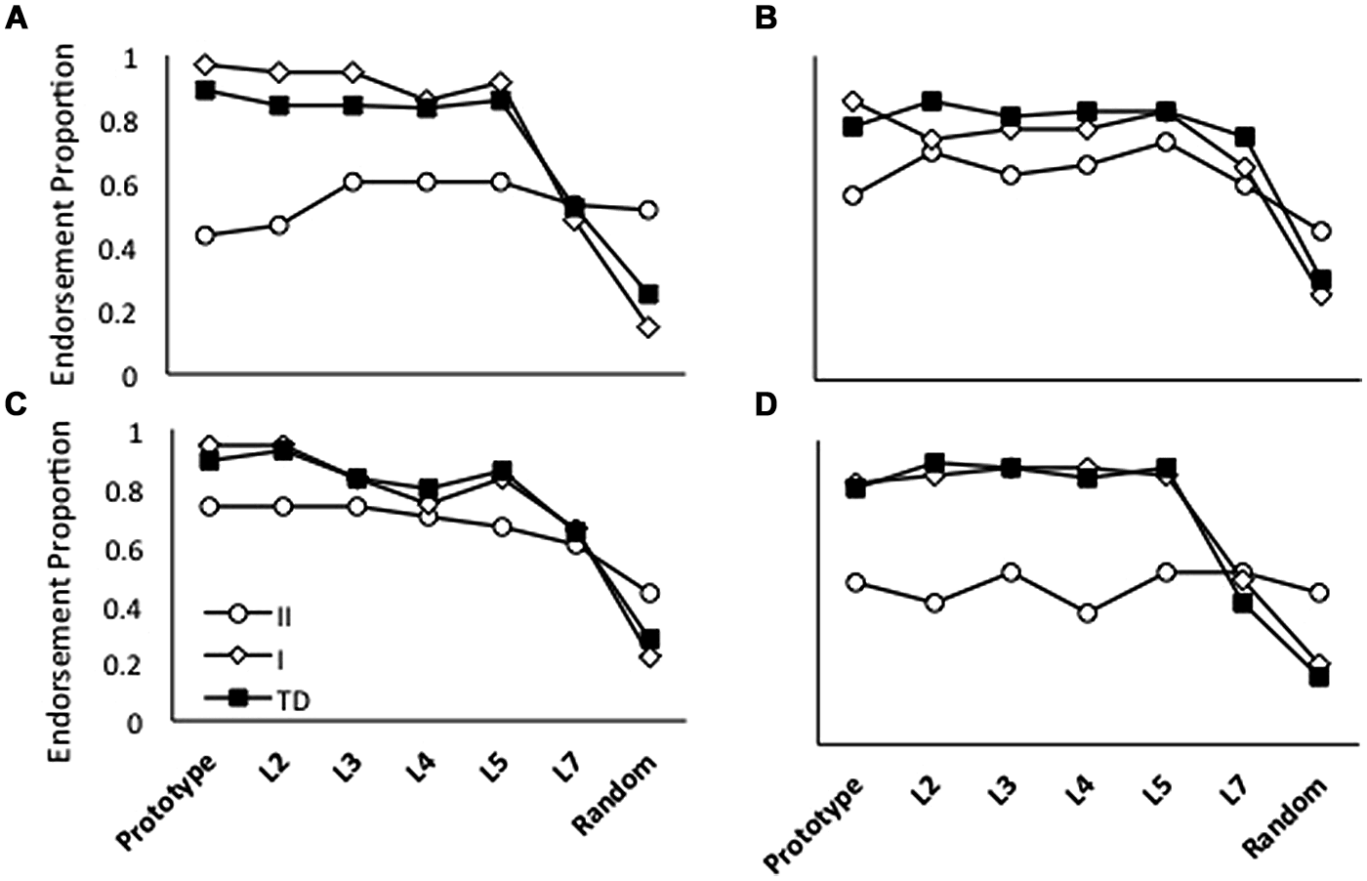
**Average endorsement proportions of Prototype, L2, L3, L4, L5, L7 and Random images during testing for 13 TD children, seven children with A Type I generalization profiles, and six children with A Type II profiles in four conditions: **(A)** Repeated; **(B)** High Distortion; **(C)** Blurry; and **(D)** Unique**.

**Figure 3** shows generalization patterns for the group of 13 children with HFASD and their matched TD controls, for each of the four different versions of training; **Figure 4** shows the same data with the HFASD children divided into the A Type I and A Type II subgroups. We conducted a 3 X (4 X 7) GLM on category endorsement (how many times participants said a stimulus belonged to the ghost category) using Child Type (A Type I, A Type II, and TD) as the between and Condition (Baseline, High Distortion, Blurry, and Unique) and Distortion Type (Prototype, L2, L3, L4, L5, L7, and Random) as the within-participant variables. We found a main effect of Child Type *F*(2,138) = 10.04, *p <* 0.001, $\eta_{p}^{2}$ = 0.27, reflecting the fact that the different groups generally endorse the category to different degrees. *Post hoc* analyses found that A Type II participants endorsed the category significantly less than either A Type I, *t*(11) = 3.74, *p* = 0.001, *d* = 0.54, or TD children *t*(17) = 4.24, *p* < 0.001, *d* = 0.92, but A Type I and TD children were not different, *t* < 1. There was also a significant main effect of Distortion Type *F*(6,138) = 49.99, *p <* 0.001, $\eta_{p}^{2}$ = 0.68, reflecting the fact that category endorsement reduced as the stimuli got increasingly distorted from the prototype. Finally, there was a significant interaction between child and distortion type, *F*(12,138) = 6.08, *p* = 0.001, $\eta_{p}^{2}$ = 0.35, again suggesting that the different groups showed different patterns of generalization. There was no significant main effect of training condition or significant interactions with training condition, all *F*’s < 2.

### Potential Individual Difference Predictors of Group Type, Variability, or Performance

In order to determine if factors like age, IQ, diagnosis, language ability, or ASD scales predict who is more likely to show the abnormal generalization patterns seen in A Type II performance, we conducted comparisons between all the children designated A Type I and A Type II on the most similar shared task (see **Table 3** for variables and means). There were no significant differences between A Type I and A Type II children on any of the measures compared, all *t*’s < 1.5. We also conducted correlation analyses to see if any of the variables correlated with overall proportion of correct endorsements or proportion of endorsement of randoms in this baseline condition. There were no significant correlations (see **Table 4**).

**Table 3:** Demographic characteristics for subgroups of HF children with ASD^a^.

	A Type I (*n* = 24)	A Type II (*n* = 25)
Characteristic	Mean (SD)	Mean (SD)
Age (years)	10.29 (1.68)	9.88 (1.62)
Parent education (years)	15.52 (2.02)	15.86 (1.52)
WISC-IV Short Form IQ	113.41 (15.49)	106.40 (13.44)
Verbal IQ (VCI)	110.18 (14.50)	106.11 (13.70)
Performance IQ (PRI)	110.27 (14.48)	107.38 (13.82)
CASL		
Expressive language	106.83 (13.59)	104.12 (15.07)
Receptive language	110.71 (13.70)	106.40 (13.36)
ADI-R		
QARSI	21.10 (4.50)	21.44 (4.58)
QAC	16.75 (3.67)	15.83 (3.73)
RRSB	6.50 (2.78)	6.30 (2.64)
	*n* (% of total)	*n* (% of total)
Gender Male	20 (83.3)	23 (92.0)
Female	4 (16.7)	2 (8.0)
Ethnicity Caucasian	21 (87.5)	24 (96.0)
African American	3 (12.5)	1 (4.0)

*^a^WISC-IV, Wechsler Intelligence Scale for Children-4th Edition; CASL, Comprehensive Assessment of Spoken Language; ADI-R, Autism Diagnostic Interview-Revised (QARSI, Qualitative Abnormalities in Reciprocal Social Interactions; QAC, Qualitative Abnormalities in Communication; RRSB, Restricted, Repetitive, and Stereotyped Patterns of Behavior). CASL and ADI-R data only collected for children in the ASD group as a screening measure for the social treatment study*.

**Table 4:** Correlations between participant variables and percent correct and percent of endorsements of random stimuli in the categorization task.

Participant variable	% Correct	% Endorsement random
Parent education^1^	0.12349	0.189449
Age^1^	0.159999	-0.09796
VCI^1^	0.123469	-0.129
PRI^1^	0.068281	-0.15688
ADIR^2^	0.064982	-0.06086
ADIR_SI^2^	0.033144	0.103619
ADIR_Com^2^	0.04056	-0.07982
ADIR_RRSB^2^	0.091041	-0.08485
CASLEXP^2^	0.04649	-0.07019
CASLREC^2^	0.070456	-0.10915

*^1^Scores are available for all the children in the study. *N* = 62, a correlation >0.25 or < -0.25 is significant. ^2^Scores are only available for the children with ASD. *N* = 49, a correlation >0.28 or < -0.28 is significant*.

Unstable performance profiles across tasks make it unlikely that stable factors like IQ, diagnosis, or ASD scales can predict A Type II performance patterns, and the comparisons and correlations confirm this. However, it is possible that these stable factors might predict variability in patterns of performance. To examine this possibility, we conducted another series of comparisons between the individual difference factors for children who switched same day tasks versus those who stayed constant (see **Table 5**). There were no significant differences, though the children who switched seemed to have a somewhat larger performance IQ (PRI), *t*(47) = 1.985, *p* = 0.053, *d* = -0.565, all other *t*’s < 1.4.

**Table 5:** Demographic characteristics of HF children with ASD who Switched A Type or Not^a^.

	Switched (*n* = 20)	Did Not Switch (*n* = 29)
Characteristic	Mean (SD)	Mean (SD)
Age (years)	10.40 (1.54)	9.86 (1.71)
Parent education (years)	15.87 (1.99)	15.57 (1.62)
WISC-IV Short Form IQ	112.08 (13.58)	108.37 (13.29)
Verbal IQ (VCI)	109.19 (14.35)	107.39 (14.16)
Performance IQ (PRI)	113.46 (13.19)	105.58 (13.90)
CASL		
Expressive language	105.90 (13.90)	105.14 (14.77)
Receptive language	108.63 (14.29)	108.71 (13.29)
ADI-R		
QARSI	20.13 (4.76)	21.96 (4.24)
QAC	16.06 (4.69)	16.37 (3.05)
RRSB	5.88 (3.28)	6.70 (2.25)
	*n* (% of total)	*n* (% of total)
Gender		
Male 17 (85.0)	26 (89.7)
Female 3 (15.0)	3 (10.3)
Ethnicity		
Caucasian 18 (90.0)	27 (93.1)
African American 2 (10.0)	2 (6.9)

*^a^WISC-IV, Wechsler Intelligence Scale for Children-4th Edition; CASL, Comprehensive Assessment of Spoken Language; ADI-R, Autism Diagnostic Interview-Revised (QARSI, Qualitative Abnormalities in Reciprocal Social Interactions; QAC, Qualitative Abnormalities in Communication; RRSB, Restricted, Repetitive, and Stereotyped Patterns of Behavior). CASL and ADI-R data only collected for children in the ASD group as a screening measure for the social treatment study*.

### Neural Network Simulations

**FIGURE 5:**
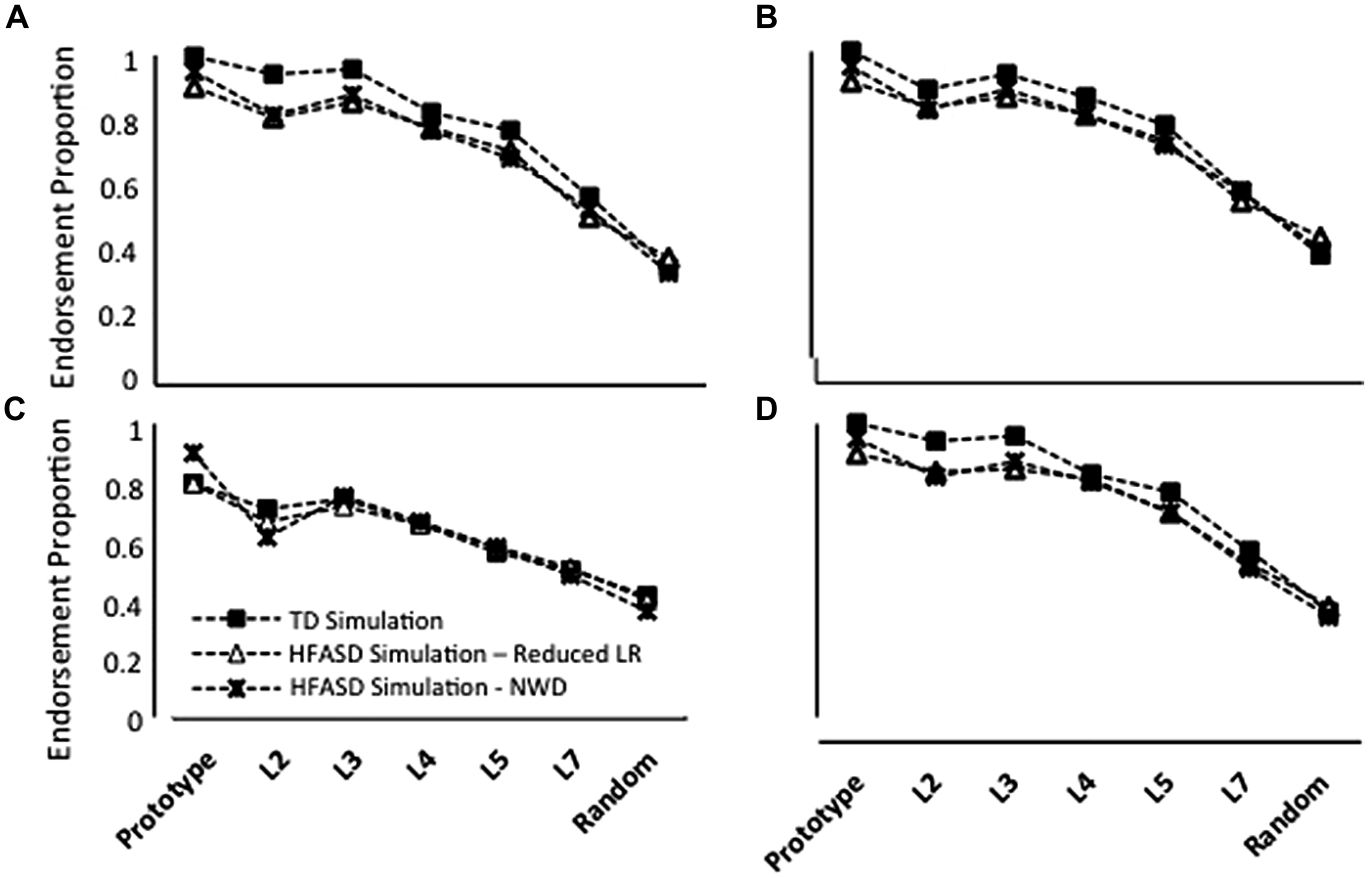
**Average endorsement proportions of Prototype, L2, L3, L4, L5, L7 and Random images during testing for 20 simulations of TD children, 20 simulations in which the overall performance of HF children with ASD was modeled using a reduced learning rate (LR) and 20 simulations in which ASD performance was modeled using negative weight decay (NWD) in four conditions: **(A)** Repeated; **(B)** High Distortion; **(C)** Blurry; and **(D)** Unique**. Note that model parameter settings were selected only for the Repeated condition and fixed for all other conditions.

In the Repeated condition, TD model endorsement rates were slightly higher than the rates produced by models with a reduced LR or NWD (**Figure 5A**). The difference between TD and ASD endorsement rates decreased as distortion level increased. False positive endorsements were approximately the same for all simulations. Note that for this training regimen, the LR or NWD parameter was chosen to qualitatively match the patterns of generalization observed behaviorally.

In the High Distortion (**Figure 5B**) and Blurry (**Figure 5C**) training conditions, endorsement rates for the TD model were also slightly higher than ASD endorsement rates, with the exception of L7 and Random endorsement rates, which were comparable between groups. Interestingly, the simulations predicted that the Blurry condition should lead to the smallest between-group differences, a prediction that was qualitatively consistent with the behavioral results (compare **Figure 5** with **Figure 3**).

Both reduced LR and NWD models produced lower endorsement rates than the TD model in the Unique training condition (**Figure 5D**), as in the Repeated condition. Again, the discrepancy betwen TD and ASD endorsements decreased with increasing distortion levels. Increasing the number or variability of training trials did not significantly affect generalization by models, as was observed behaviorally.

Endorsements associated with A Type II generalization were so low (**Figure 4**) that untrained NNs produced a generalization pattern that roughly approximated the pattern seen behaviorally. However, the overall endorsement rates of these networks were consistently lower than those associated with the A Type II profile. Training networks for a single epoch at a LR of 6.00E-04 produced endorsement rates comparable to the A Type II profile at all stimulus levels (although the combination of low LR and little training led to high variability in generalization across networks). Using a single epoch of training, a reasonable approximation to A Type I/TD generalization was produced with a LR of 0.1. Combining the two NN generalization profiles using a weighted average in which there were slightly fewer A Type II models (*N* = 6) than A Type I models (*N* = 7) produced a group-level generalization pattern similar to that observed in the current behavioral experiment in which the prototype was endorsed at levels comparable to (or slightly less than) distorted prototypes. Thus, the two-subgroup instantiation of the connectionist model provided a better approximation of the atypical generalization patterns observed in HF children with ASD than was possible with models that assumed a uniform performance pattern across these children (replicating the results of Dovgopoly and Mercado, 2013).

## Discussion

The current results confirm the findings of Church et al. (2010, 2015) that subsets of HF children with ASD show degraded learning and generalization when trained to categorize novel abstract shapes. When children with ASD ran into difficulties, they showed much less generalization than was shown by TD children. The “subgroups” of children with ASD who showed category-learning deficits did not differ systematically in terms of IQ measures, diagnostic classifications, or language abilities from those who performed like TD children. In fact, when required to perform the same category-learning task using slightly different shapes, many children with ASD switched from being atypical performers to typical performers or vice versa. Such fluctuations in performance may partly account for the mixed findings regarding category-learning deficits in individuals with ASD.

### Heterogeneity in Visual Category Learning and Generalization

The current data show that HF children with ASD who are faced with identical visual category learning tasks can differ dramatically in what they learn (see also Molesworth et al., 2008; Church et al., 2010, 2015; Charman et al., 2011; Froehlich et al., 2012; Schipul, 2012). What distinguishes the child with ASD who easily learns a perceptual category from one who runs into problems? To date, no specific cognitive correlate has been identified that reliably predicts which individuals with ASD will have problems learning perceptual categories (Church et al., 2010; Vladusich et al., 2010; Soulières et al., 2011). Perhaps the simplest explanation for the observed variations in performance is that some children with ASD were less engaged during training or testing (i.e., children with ASD who were not engaged by the task performed poorly). Although this interpretation is difficult to rule out based on behavioral observations alone, it fails to explain why so many children and adults with ASD across multiple studies are not engaged by category-learning tasks when TD individuals have no problems performing those same tasks. Nor does it explain why when HF children with ASD are showing abnormal learning, they are not showing more behavioral manifestations of inattention, distraction, or lack of engagement than when their learning is comparable to TD children. Furthermore, to account for the dichotomous generalization profiles shown here and in earlier work (Church et al., 2010, 2015), one would have to assume that HF children with ASD rarely show intermediate levels of task engagement when learning to categorize abstract shapes, because these children did not show intermediate performance levels.

Various factors might lead a child to perform poorly in a computer-based category-learning task. A child’s attention might wander during the training session. Some children might misunderstand the instructions or might fixate on features of shapes that are irrelevant to task performance. Others might have specific cognitive deficits, such as executive dysfunction, that interfere with task learning. Such factors provide plausible *post hoc* accounts of why subsets of children (and adults) with ASD might have difficulty learning visual categories. Importantly, however, such accounts fail to explain why subsets of individuals with ASD are intermittently more prone to such problems than are TD individuals. If a child with ASD has impaired executive functions or perceptual abilities, then it is unclear why those dysfunctions would negatively affect category learning for some shapes, but not others. Similarly, if a child is able to successfully learn to categorize shapes in one task, it seems unlikely that any confusion about instructions would arise when they are later asked to perform the same task with different shapes. Given the similarity of atypical generalization profiles across multiple category learning tasks and participant samples, it seems likely that common issues are leading to difficulties in many children with ASD. What those specific issues are remains unknown, but the current evidence suggests that they are not omnipresent differences in executive control, task understanding, social skills, or perceptual biases.

Past investigations of visual category learning by individuals with ASD have focused on determining how well these individuals learn to perform various categorization tasks relative to TD individuals (Klinger and Dawson, 2001; Bott et al., 2006; Molesworth et al., 2008; Gastgeb et al., 2009, 2012; Church et al., 2010; Vladusich et al., 2010; Soulières et al., 2011; Froehlich et al., 2012). Results from the current study show that for HF children with ASD, within-individual variations in category learning performance can be as large as those observed between children with and without ASD. Essentially what this means is that a child with ASD might show considerable learning and generalization after training with one set of abstract shapes, little generalization when subsequently trained to categorize different abstract shapes, and typical learning and generalization when trained with a third or fourth set of shapes. Consequently, the A Type I and A Type II classifications apparently do not distinguish subgroups of children with ASD, but instead correspond to two characteristic performance profiles, both of which could potentially be shown by a single child with ASD within a single experimental session. The factors that might lead a child with ASD to switch from atypical learning and generalization to more typical learning (or vice versa) are unknown. Future studies examining within-individual variations in category learning for both children and adults with ASD are needed to better understand when and why difficulties in category learning and generalization arise.

Within-individual variations in category learning and generalization by children with ASD might reflect a particular stage or mode of cognitive development. For example, when TD children learn new mathematical skills, their performance can fluctuate dramatically across problems that differ only slightly (Siegler, 1987, 1996). It is not known when category-learning abilities are fully developed in either TD children or in children with ASD, making it difficult to determine when such variability might be present in either group. There have been no studies of category learning in younger children diagnosed with ASD. Longitudinal studies involving repeated training and testing of multiple category learning tasks by children with ASD beginning during the pre-school years are critical to assessing the prevalence and consistency of category learning deficits. Experiments on adults may underestimate the prevalence of category learning deficits in children with ASD, because adults are familiar with a larger number and variety of categories that can potentially help them to learn new categories.

To our knowledge, no existing models of category learning by children with ASD can predict or explain the dramatic within-individual fluctuations in performance observed in the current study. One could question whether it is the category learning performances of the children that are varying, or whether the methods used to measure their performance are simply unreliable. The category learning tasks used in the current study were selected because other researchers have used these tasks extensively over several decades. If these methods give reliable results for TD individuals, but not for individuals with ASD, then this would still indicate that category learning processes differ between these two groups in some way that has yet to be explained. In the following, we evaluate possible explanations for these findings in the context of current theories of category learning as well as neural mechanisms that might give rise to atypical learning, perception, and generalization in individuals with ASD.

### Implications for Current Neurally Based Theories of ASD

Neurally based accounts of perceptual processing deficits in ASD have pointed to effects of atypical cortical connectivity (Just et al., 2004, 2012; Kana et al., 2011), degraded functioning of the dorsal/magnocellular system (Spencer et al., 2000), minicolumn pathology (Casanova et al., 2002, 2006), and disrupted neural excitation and inhibition (Rubenstein and Merzenich, 2003; Yizhar et al., 2011), as likely sources of dysfunction. More generally, neuroscientists have proposed that many of the behavioral symptoms associated with ASD result from cortical dysfunction (Rubenstein and Merzenich, 2003; Markram and Markram, 2010; LeBlanc and Fagiolini, 2011), and abnormal synaptic function (Ramocki and Zoghbi, 2008; Bourgeron, 2009; Auerbach et al., 2011; Schmeisser et al., 2012). None of these theories predicts or explains why subsets of children with ASD might differ dramatically in their ability to learn perceptual categories, and all implicitly predict that if a child with ASD shows deficits in learning to categorize abstract shapes, then training the child with different shapes or training regimens is unlikely to overcome this deficit.

Proponents of neurally based theories of ASD-related deficits generally focus on explaining how abnormalities in various brain regions contribute to core symptoms, providing only broad suggestions about why children with ASD are so behaviorally heterogeneous (Rubenstein and Merzenich, 2003; Grossberg and Seidman, 2006; Markram and Markram, 2010; Just et al., 2012). Past attempts to link dysfunction in specific brain regions to specific cognitive deficits seen in ASD have led to mixed results, with some investigators reporting structural abnormalities in various regions, and others reporting that those same regions do not differ from what is seen in TD individuals (Waterhouse et al., 1996; Penn, 2006; Markram and Markram, 2010; Schroeder et al., 2010; Lenroot and Yeung, 2013; Waterhouse, 2013). Grossberg and Seidman (2006) argued that the involvement of multiple, abnormally functioning brain regions during development leads to the behavioral heterogeneity associated with ASD (see also Lee et al., 2003). Just et al. (2012) similarly suggested that the heterogeneity of atypical neural connections accounted for symptomatic heterogeneity. Rubenstein and Merzenich (2003) attributed ASD-related behavioral heterogeneity to the heterogeneity of underlying genetic factors (see also Folstein and Rosen-Sheidley, 2001; Jeste and Geschwind, 2014). Individuals with ASD do show heterogeneous patterns of neural connectivity and activity (Salmond et al., 2007; Byrge et al., 2015; Hahamy et al., 2015), consistent with these proposals. Such neural variability likely contributes to individual differences in symptoms and might also lead to differences in category learning abilities. However, neither genetic nor neural heterogeneity adequately accounts for why a child with ASD might show typical category learning capacities for some shapes but not others.

Simulations using an existing NN model of visual object recognition (Henderson and McClelland, 2011) suggest that atypical category learning and generalization may reflect dysfunctional neural plasticity or homeostasis (Dovgopoly and Mercado, 2013). Several other computational models have been developed to simulate the effects of ASD-related neural abnormalities on behavior (O’Laughlin and Thagard, 2000; Bjorne and Balkenius, 2005; Grossberg and Seidman, 2006; Noriega, 2007; Thomas et al., 2011; Just et al., 2012), but no other model predicts the quantitative outcomes of particular category learning tasks. Dovgopoly and Mercado’s (2013) NN model successfully predicted how children with ASD would generalize when trained to categorize specific sets of abstract shapes (Church et al., 2015), as well as how children would generalize when trained using different regimens (current study). Despite these successes, the NN model can only account for within-individual fluctuations in category-learning capacity by introducing the auxiliary assumption that neural plasticity varies greatly over relatively short periods in HF children with ASD. This assumption is consistent with past findings of dysfunctional cholinergic modulatory systems in the basal forebrains of individuals with ASD (Perry et al., 2001; Riva et al., 2011; Suzuki et al., 2011). However, given the dearth of data on the dynamics of basal forebrain activity in children with or without ASD, such an assumption must be viewed as a highly speculative prediction/hypothesis.

## Conclusion

The current findings suggest an alternative explanation for why past studies of category learning by individuals with ASD have produced such mixed results. Namely, individuals with ASD may be much more sensitive to the specific experimental conditions used in category learning experiments than are TD individuals, and the conditions that disrupt or facilitate category learning may vary idiosyncratically across individuals with ASD. Heterogeneity in the capacities and sensitivities of individuals with ASD is not specific to category learning and can be observed in social impairment (Waterhouse, 2013), as well as in physiological responses (e.g., Hirstein et al., 2001). Although such heterogeneities in deficits within and across individuals with ASD are widely recognized by researchers, the possibility that comparable performance variations might also be present within particular cognitive capacities does not appear to have been examined or discussed in past work. Refinements in experimental design that take into account possibly large, systematic variations in performance by children with ASD are needed to better understand how neural abnormalities contribute to the development of heterogeneous symptoms (Georgiades et al., 2013).

Our results highlight the importance of theoretical guidance when developing interventions that aim to facilitate learning and generalization in children with ASD. The current findings indicate that it may be quite difficult to predict when a particular child with ASD will run into difficulties forming categories from repeated experiences, and that events that a typical child might readily perceive as being similar to or different from past experiences might not be perceived similarly by a child with ASD. Conversely, a child with ASD might be acutely aware of differences between stimuli or events that a TD child or adult would not notice, which might significantly affect what that child learns about the world. Given that many fundamental perceptual and conceptual categories are formed during early development, understanding how, when, and why category learning and generalization processes fail in children with ASD may prove crucial to understanding how the negative effects of ASD might best be circumvented. An important lesson from the current study is that children with ASD who run into difficulties in a particular learning context may show unsuspected capacities when given the opportunity to learn the same skill under slightly different circumstances.

## Conflict of Interest Statement

The authors declare that the research was conducted in the absence of any commercial or financial relationships that could be construed as a potential conflict of interest.

## Footnotes

1 Analyses done separately with just the 26 children from Situation A still found no main effects of or interactions with order or stimulus set. This suggested that even in the longest experimental sessions fatigue did not seem to play a significant role.

## ABBREVIATIONS

ADI-R: Autism Diagnostic Interview-Revised
ASD: autism spectrum disorder
CASL: Comprehensive Assessment of Spoken Language
HF: high functioning; LR, learning rate
NWD: negative weight decay
QAC: Qualitative Abnormalities in Communication
QARSI: Qualitative Abnormalities in Reciprocal Social Interactions
RRSB: Restricted, Repetitive, and Stereotyped Patterns of Behavior
SOM: self organizing map
TD: typically developing
WISC-IV: Wechsler Intelligence Scale for Children-4th Edition
