# Supplementary material for: Heterogeneity in perceptual category learning by high functioning children with autism spectrum disorder
Source: Front Integr Neurosci. 2015 Jun 23;9:42. doi: 10.3389/fnint.2015.00042 (PMC4477144; doi:10.3389/fnint.2015.00042)
Supplement: Supplementary file 1 [file Data_Sheet_1.DOCX]

**Supplementary Materials**

**Stimuli**

The abstract shape stimuli were created using a Turbo 7 computer program. This program generates a number of stimuli from a prototype. This program generates these stimuli by first selecting nine dots in a central 30 x 30 area of a 50 x 50 grid of pixels. A successive (in the order randomly chosen) joining of the first nine dots by lines form the prototype. The distortions of the prototype are created by varying the probability that dots can move in a five-area space (Area 1, Area 2, Area 3, Area 4, and Area 5). In area 1, the dot does not move. In area 2, the dot moves to one of the 8 pixel positions around the original stimulus. In area 3, the dot moves to one of the 16 pixel positions which are located around Area 2. In Area 4, the dot moves into one of the 75 pixel positions in the third, fourth, and half of the fifth pixel shell around Area 1, 2, and 3. In Area 5, the dot moves into one of the last 300 pixel positions in the surrounding 20 x 20 pixel grid.

By applying different probabilities to each area, the program can create different levels of distortion. For example: highly distorted stimuli are likely to have high probabilities in Area 4 and 5. Table S1 illustrates the probability of each area for all level of distortions.

Stimuli that are not members of the category are called Random. These Random stimuli are created by generating L7 distortions of prototypes from an unrelated category.

Dot-distortion stimuli were magnified three times their original size to be more visible to participants. We increased the size of these stimuli by mapping each point to a 3x3 pixel area and adding the point to the center of this area. So, the points were now drawn in a 150 by 150 area space. Lastly, we used the procedures DrawPoly and FillPoly from Turbo Pascal 7.0 to connect the dots with line and fill the now polygon with color. The DrawPoly procedure draws the outline of a polygon. The code used in the program is the following: DrawPoly (9, NormedObject). 9 is the number of coordinates included in NormedObject. NormedObject includes the values of each coordinate (X,Y) for each intersection in the polygon. The FillPoly procedure fills the polygon. The code used in the program is the following: FillPoly(9, NormedObject). 9 is the number of coordinates included in NormedObject. NormedObect includes the values of each coordinate (X,Y) for each intersection in the polygon (Hennefeld,1994).

Table S1

Distortion Level Area 1 Area 2 Area 3 Area 4 Area 5
